# Investigating metabolic interactions in a microbial co-culture through integrated modelling and experiments

**DOI:** 10.1016/j.csbj.2020.03.019

**Published:** 2020-03-30

**Authors:** Aarthi Ravikrishnan, Lars M. Blank, Smita Srivastava, Karthik Raman

**Affiliations:** a Department of Biotechnology, Bhupat & Jyoti Mehta School of Biosciences, Indian Institute of Technology (IIT) Madras, Chennai 600 036, India; b Initiative for Biological Systems Engineering, IIT Madras, India; c Robert Bosch Centre for Data Science and Artificial Intelligence, IIT Madras, India; d Institute of Applied Microbiology - iAMB, Aachen Biology and Biotechnology – ABBt, Worringer Weg 1, RWTH Aachen University, D-52074 Aachen, Germany

**Keywords:** Microbial interactions, Metabolic exchanges, Metabolic Support Index, Microbial co-cultures, Pathway analyses

## Abstract

Microbial co-cultures have been used in several biotechnological applications. Within these co-cultures, the microorganisms tend to interact with each other and perform complex actions. Investigating metabolic interactions in microbial co-cultures is crucial in designing microbial consortia. Here, we present a pipeline integrating modelling and experimental approaches to understand metabolic interactions between organisms in a community. We define a new index named “Metabolic Support Index (MSI)”, which quantifies the benefits derived by each organism in the presence of the other when grown as a co-culture. We computed MSI for several experimentally demonstrated co-cultures and showed that MSI, as a metric, accurately identifies the organism that derives the maximum benefit. We also computed MSI for a commonly used yeast co-culture consisting of *Saccharomyces cerevisiae* and *Pichia stipitis* and observed that the latter derives higher benefit from the interaction. Further, we designed two-stage experiments to study mutual interactions and showed that *P. stipitis* indeed derives the maximum benefit from the interaction, as shown from our computational predictions. Also, using our previously developed computational tool MetQuest, we identified all the metabolic exchanges happening between these organisms by analysing the pathways spanning the two organisms. By analysing the HPLC profiles and studying the isotope labelling, we show that *P. stipitis* consumes the ethanol produced by *S. cerevisiae* when grown on glucose-rich medium under aerobic conditions, as also indicated by our *in silico* pathway analyses. Our approach represents an important step in understanding metabolic interactions in microbial communities through an integrated computational and experimental workflow.

## Graphical abstract

**Figure:**
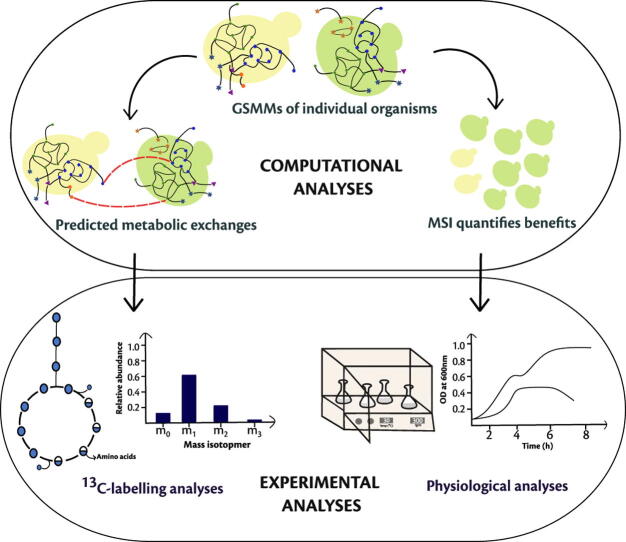

## 1 Introduction

Microbial co-cultures have been broadly used in several biotechnological applications, owing to their abilities to produce a broader pool of enzymes, which enable the degradation or synthesis of complex molecules. They exhibit division of labour and can be used to explore the joint metabolic capabilities of the constituent organisms. Microbial co-cultures have been employed to carry out complex functions ranging from xenobiotic degradation [1] to synthesising novel secondary metabolites [2], [3]. More recently, the application of co-culture systems to produce biofuels has also been gaining traction, where groups of microorganisms, either wild-type or engineered, have been employed to convert lignocellulosic biomass to ethanol [4], [5], [6].

Besides, there have also been advances in engineering microbial co-cultures to produce fine chemicals. Such synthetic microbial communities consist of organisms that have been engineered to communicate with each other via metabolic exchanges. For instance, in one such study [7], *Escherichia coli* cells were manipulated by engineering pathways to exchange metabolites for improving the titres of *n*-butanol. In a few other studies [8], [9], separate *E. coli* cells were engineered with specialised metabolic pathways to communicate with one another through metabolic exchanges. This engineered *E. coli* co-culture was used for the production of industrially essential chemicals such as *cis,cis*-muconic acid, 3-aminobenzoic acid and resveratrol.

In addition to industrial applications, co-cultures are also useful to understand interactions between organisms. In a community, microbes orchestrate several complex functions by communicating with one another, commonly via metabolic interactions [10]. These interactions define the relationships between the organisms and shape the overall structure of the microbial community. Computational studies have provided insights into the pairwise relationship between members in a community [11], [12], [13], [14], [15].

Understanding the metabolism of individual organisms, as well as investigating the metabolic interactions that happen in a community, is central to designing a microbial consortium for a given application. There are likely multiple metabolites exchanged between the consortium members. Experimentally identifying and understanding the role of these metabolic exchanges and interactions entails the profiling of both intracellular and extracellular metabolites. Such untargeted metabolomic profiling becomes very difficult [16], especially when it involves a co-culture of organisms. Although complete exometabolomic analysis has been recently carried out for a few microbial communities [17], [18], it is still very difficult to map the individual metabolites to the organisms that produced them in the co-culture.

In this study, we design a comprehensive workflow integrating computational and experimental approaches to predict and validate the microbial interactions in a co-culture. We adopt a three-pronged approach to study a co-culture of two industrially important yeast species [19], viz., *Saccharomyces cerevisiae* and *Pichia stipitis*, drawing on computational analyses, physiological studies and ^13^C-labelling experiments. First, using our previously developed graph-theoretic algorithm, MetQuest [20], we quantify the possible benefits derived by the microorganisms when they stay together in a community. We propose a new metric called *Metabolic Support Index (MSI)* to determine which organism derives a relatively higher benefit from the interaction. We show that this metric can successfully quantify the interactions between the organisms using a few examples of previously demonstrated microbial co-cultures. Using one of the widely used co-cultures consisting of *S. cerevisiae* and *P. stipitis*, we test the various predictions from our computational analyses. Several studies previously performed have used these organisms not only to produce ethanol from multiple carbon sources [21], [22], [23] but also to computationally model and understand the interactions between the organisms [24], [6]. With this model system of organisms, we identify potential metabolic exchanges and perform experimental verifications. Next, using isotope-labelling studies, we highlight the metabolic interactions between the organisms. The results from our workflow on this model co-culture indicate that *P. stipitis* benefits from the interaction and takes up the ethanol produced by *S. cerevisiae* when grown on glucose-rich medium under aerobic conditions. Our approach represents an important step in integrating modelling with experiments to understand and characterise microbial interactions in communities. Ultimately, the design of such microbial communities for specific applications is envisaged.

## 2 Materials and methods

### 2.1 Computational methods

In this section, we describe the *in silico* methods to calculate the *Metabolic Support Index (MSI)* and identify the metabolic exchanges between the organisms.

#### 2.1.1 Calculation of MSI

**Fig. 1:**
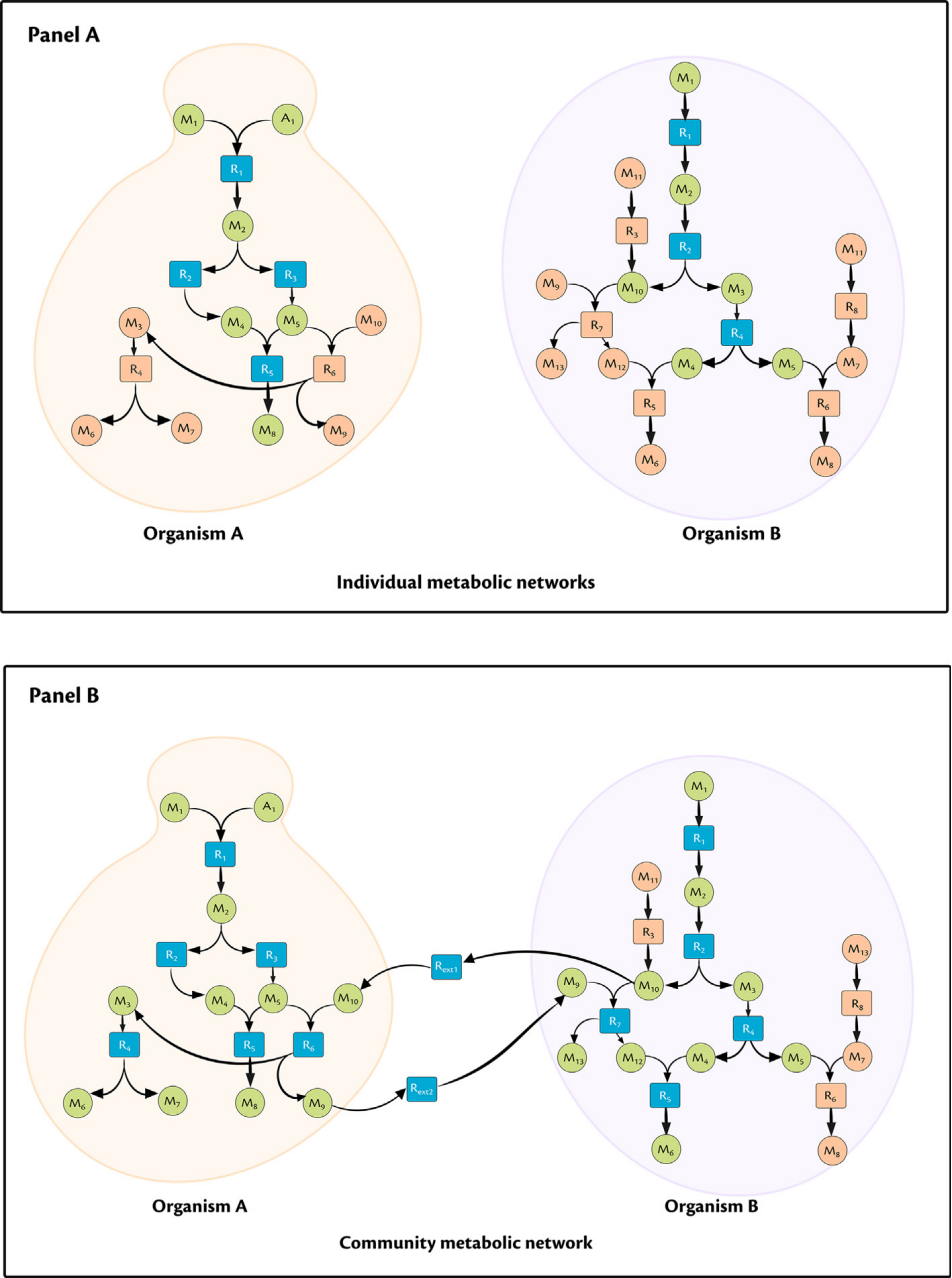
Metabolic Support Index (MSI) calculations. (I) Bipartite graph of individual organisms. (II) Community metabolic network showing the metabolic exchanges. Circular nodes are metabolites (eg $M_{1},M_{2}$), square nodes are the reactions (eg $R_{1},R_{2}$), green coloured circular nodes represent the metabolites in the scope of $M_{1}$ and $A_{1}$, orange coloured circular nodes represent the metabolites that is not present in the scope, orange coloured square nodes represent the stuck reactions, blue coloured square nodes represent the visited nodes. Here we see that $n_{\mathrm{stuck},A|A}$ = 2, $n_{\mathrm{stuck},A|A\cup B}$ = 0, MSI(A|A∪B) = 1; similarly $n_{\mathrm{stuck},B|B}$ = 5, $n_{\mathrm{stuck},B|A\cup B}$ = 3, MSI(B|A∪B) = 0.4. Thus, we see that the Organism *A* benefits more from this interaction with Organism *B*. (For interpretation of the references to colour in this figure legend, the reader is referred to the web version of this article.)

To quantify the benefits derived by a given organism in a community, we define a new metric, Metabolic support index (MSI). We compute MSI for a given organism *A* in the community A∪B, as the fraction of reactions *stuck* in the metabolic network of *A*, but relieved in the presence of organism *B* in the community (see Fig. 1) as

(1) $$\mathrm{MSI}(A|A\cup B)=1-\frac{n_{\mathrm{stuck},A|A\cup B}}{n_{\mathrm{stuck},A|A}}$$

where A∪B denotes the community metabolic network comprising both organisms *A* and B,A denotes the bi-partite metabolic network of organism A, $n_{\mathrm{stuck},A|A}$ denotes the number of stuck reactions in $A,n_{\mathrm{stuck},A|A\cup B}$ denotes the number of stuck reactions of *A* in A∪B. Further, MSI(A|A∪B)=1 indicates that the organism *A* fully benefits from the interaction with organism B,MSI(A|A∪B)=0 indicates that organism *A* does not derive any benefit from the interaction with organism *B*. It should be noted that not all the reactions that have been activated would be used by the organism for the growth. MSI indicates the increase in the metabolic capacities of the organisms and does not merely capture the support an organism receives for higher growth.

We used our previously developed algorithm, MetQuest [20], to determine the number of reactions that can be *visited* or *stuck*, depending on the presence of precursor metabolites. *Stuck* reactions are those reactions whose precursor metabolites cannot be synthesised by the metabolic network using the given input conditions, while *visited* reactions are those reactions whose input metabolites are all present and hence the reaction can proceed.

To calculate MSI for a microorganism, we first constructed the directed bipartite graph of the individual organism, followed by the joint bipartite graphs of the combination of organisms from their respective genome-scale metabolic models (GSMMs) using the construct_graph module from the previously developed metquest Python package (https://github.com/RamanLab/metquest). Depending on the availability of the GSMMs of the organisms, we obtained them either from the respective publications [25], [26], [27], [28], [29] or from the Path2Models database [30]. We applied our algorithm using a set of starting seed metabolites, which included the co-factors, co-enzymes and the carbon source used in the respective experiments. For our co-culture of interest, i.e., *S. cerevisiae* and *P. stipitis*, the seed metabolite set consisted of components from YNB minimal medium, D-glucose as the carbon source along with the set of co-factors and co-enzymes. All the data files are available in Supplementary data.

Next, using the guided_bfs module in the package, we obtained the number of *stuck* reactions from two different scenarios: (a) when the organisms are analysed independently as “single” graphs ($n_{\mathrm{stuck},A|A}$), (b) when they are grown along with other organisms as a “joint” graph ($n_{\mathrm{stuck},A|A\cup B}$). Using Eq. (1), we calculated the MSI of the organisms. Also, in both the cases, we also obtained the *scope*, i.e., the set of metabolites that can be produced from the given set of seed metabolites. All the simulations were carried out in Python 3.6 on an Intel®Core i7-2600 Desktop with 24 GB RAM, running Ubuntu 18.04.1 LTS.

#### 2.1.2 Pathway analyses on the community metabolic network

To identify the potential metabolic exchanges happening between *S. cerevisiae* and *P. stipitis*, we first enumerated all the pathways until a pathway length cut-off 75 using the find_pathways function in MetQuest package. We constructed the joint bipartite graph of *S. cerevisiae* and *P. stipitis* from their respective GSMMs [29], [27] using the construct_graph module. We used the components of YNB medium as seed metabolites along with D-glucose as the carbon source. Using home-grown Python scripts, we analysed every pathway from *S. cerevisiae* that lead to every scope metabolite in *P. stipitis* for the presence of metabolites from *S. cerevisiae*. We carried out these simulations on an Intel®Xeon®CPU E7-4850 v4 @ 2.10 GHz workstation with 1 TB RAM, running CentOS 7.5.1804. The scripts and the data used in these analyses are available in online datasets (Supplementary data).

### 2.2 Experimental methods

#### 2.2.1 Yeast strains used

In this study, we used *S. cerevisiae* (MTCC 171) and *P. stipitis* (NCIM 3497), procured from Microbial Type Culture Collection, Chandigarh, India, and National Collection of Industrial Microorganisms, Pune, India, respectively.

#### 2.2.2 Culture maintenance and inoculum preparation

The yeast strains were independently maintained on YPD agar consisting of 3 g/L Yeast extract, 10 g/L Peptone, 20 g/L Glucose and 1% agar (HiMedia Laboratories Pvt Ltd, Mumbai, India). From the agar plate, one colony was picked and inoculated in YPD medium and was grown at 30 °C. For the long-term storage, cultures from mid-log phase were collected and maintained as 30% (v/v) glycerol stocks and stored at −80 °C.

Glycerol stocks of the respective yeast strains were revived by streaking them onto a YPD Agar plate and incubating at 30 °C for 24 h. The primary cultures were initiated as suspension cultures by inoculating single colony on YPD medium, followed by YNB minimal medium without amino acids (Sigma–Aldrich, Germany) supplemented with 10 g/L glucose (Carl Roth GmBH, Germany). The culture was grown in minimal medium until mid-logarithmic phase and used for subsequent experiments. All the experiments were carried out in YNB minimal medium using glucose (Carl Roth GmBH, Germany) as the carbon source.

#### 2.2.3 Growth kinetics of mono-culture and co-culture

To determine the substrate utilisation and product secretion profiles, growth kinetic experiments of both the yeast strains, *S. cerevisiae* and *P. stipitis* were carried out. The culture from the well-grown primary inoculum was inoculated in 500 mL Erlenmeyer flasks consisting of 25 mL of YNB medium with 10 g/L Glucose. For all the experiments, we maintained the starting Optical Density (OD) as 0.17. The experiment was carried out at 300 rpm, 30 °C and the growth was continuously monitored online using the Cell Growth Quantifier (CGQ; Aquila Biolabs GmBH, Germany). Samples were withdrawn at regular intervals for analysing the spent metabolites through High-Performance Liquid Chromatography (HPLC).

#### 2.2.4 Growth analysis in the spent medium

We designed a two-step shake flask experiment to determine if there were any interactions between the organisms. Briefly, in the first stage, *S. cerevisiae* and *P. stipitis* were independently grown in minimal YNB medium with 10 g/L Glucose. The growth was continuously monitored online using CGQ (Aquila Biolabs GmBH, Germany), and the samples were withdrawn at the start of the experiment, early and late exponential phase. CGQ measures the backscattered light emitted by the growing microbial cells. Data analysis, processing and visualisation, were carried out using CGQuant software version 7.3 (Aquila Biolabs GmBH, Germany).

**Fig. 2:**
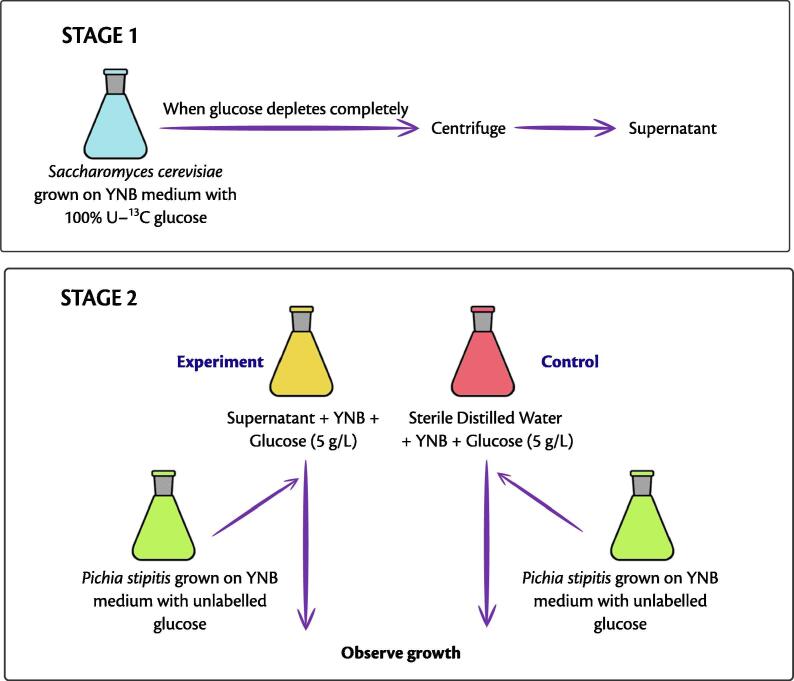
Two-stage experimental setup to identify metabolic interactions. In stage 1 (top panel), the first organism is grown in a minimal medium consisting of glucose. In stage 2 (bottom panel), the supernatant from the first organism is used as the medium to grow the second organism, and the growth is compared with that of the control where sterile distilled water is used instead of the supernatant.

Samples were analysed in HPLC to check if the carbon source was completely depleted. At this stage, the cells were separated from the broth by centrifuging at 8000 rpm, 4 °C for 10 min, and the supernatant was filter sterilised. In the next step, to these supernatants, YNB medium and 5 g/L glucose were added. This was done to ensure that these organisms could grow and build the necessary machinery to take up the nutrients from the supernatant. The working volume was 20 mL. We designated the spent medium obtained from *S. cerevisiae* and *P. stipitis* as *SupSce* and *SupPst* respectively. We inoculated these supernatants with either of these organisms, i.e., to the *SupSce* we added *P. stipitis* and to the *SupPst* we added *S. cerevisiae*, such that the initial OD was 0.17±0.1. We continuously monitored the growth of these organisms using CGQ and analysed the samples for residual carbon source and the extracellular metabolites using HPLC at the start and end of experiments. Also, we compared the growth of the organisms with that in the control, where the supernatant was replaced with sterile distilled water. The experimental scheme is as shown in Fig. 2.

To check for the metabolic interactions, we repeated the same two-stage experiments as above, with a single modification. Instead of naturally labelled glucose, we used 100% uniformly labelled (U-^13^C) glucose as the carbon source in the first step to cultivate the microorganisms individually. Samples withdrawn were analysed through HPLC for the residual carbon and the extracellular metabolites. The biomass at the end of 24 h was analysed using Gas chromatography-mass spectrometry (GC–MS) to identify the labelling patterns of the amino acids alanine, valine, serine and aspartate.

### 2.3 Analytical methods

#### 2.3.1 Quantification of glucose and other by-products

The yeast supernatant was quantified using High Performance Liquid Chromatography (HPLC) (System Gold125 Solvent Module, Beckman Coulter, USA) with an organic acid stationary phase (300×8 mm, 10 μm particle size) (CS Chromatographie Service GmbH, Langerwehe, Germany) under the following conditions: Mobile phase 5 mM Sulfuric acid, Flow rate – 0.5 mL/min, Column Temperature – 50 °C using a Refractive Index Detector (KNAUER Wissenschaftliche Geráte, Berlin, Germany). Standard plots (with R^2^ = 0.98) for the metabolites ethanol (Carl Roth GmbH, Germany) (0.25–2 % (v/v)) and glucose (2–15 g/L) were prepared by injecting known quantities and measuring the area under the respective peaks.

#### 2.3.2 GC–MS to identify labelling patterns in biomass

To identify the labelling patterns in the biomass, we adapted the protocol from [31]. Briefly, 0.3–0.4 mg of the biomass were resuspended in 150 μL 6 M Hydrochloric acid (HCl) and transferred to 1.5 mL glass vials (Part Number AR0-3940-12 Phenomenex). The suspension was incubated at 105 °C for 6 h for hydrolysis, and dried overnight at 85 °C. To the dried hydrolysate, 30 μL acetonitrile and 30 μL N-methyl-N-tert-butyldimethylsilyl-trifluoroacetamide (MBDSTFA) was added and incubated at 85 °C for 1 h. The samples were cooled and immediately analysed using a GC–MS single quadrupole system using the “Full scan” mode. The system consisted of a TRACE^TM^ GC Ultra, a TSQ 8000 XLS Triple-Quadrupole MS equipped with PTV-injector (Thermo Fisher Scientific, Waltham, MA, USA) and a ThermoScientific TriPlus RSH Autosampler. The separation of the amino acids was achieved using Trace GOLD TG-SilMS fused silica column (length 15 m; inner diameter 0.25 mm; film thickness 0.25 μm). The injector temperature was set at 270 °C, and the column oven was set at 140 °C for 1 min, and the temperature was steadily increased to 310 °C with a ramp of 10 °C/min, and a hold time of 1 min. The equipment was operated under a steady gas flow of 1 mL/min of helium with a split ratio of 1/15. For every measurement, 1 μ L of the sample was injected. The resulting chromatogram and the mass spectra of the samples were analysed by comparing with those of amino acids standards (Sigma Aldrich, Germany). All the mass spectra and the chromatograms were analysed on Thermo XCalibur 2.2 software (Thermo Fisher Scientific, Waltham, MA, USA).

#### 2.3.3 *In silico* methods to identify the isotopomer distribution in amino acids

From the chromatogram and the mass spectra, the peaks were identified by comparing the retention times of different amino acids with that of standards. The mass spectrum of each derivatised amino acid was also compared against the NIST Library using NIST MS Search software (https://www.nist.gov/srd/nist-standard-reference-database-1a-v17) to confirm the presence of corresponding amino acid. Also, we determined the average carbon labelling and the relative abundance of every fragment after performing corrections for proton gain and original biomass using the iMS2Flux software [32]. For all our calculations, we used the (M-57) fragment of the amino acids, since it captures information about all the carbon atoms in an amino acid.

## 3 Results

In this study, we present a workflow integrating modelling and experimental approaches to understand the microbial interactions between organisms in a community. In the current section, we present the results from our three-pronged approach, which includes computational, physiological and labelling studies.

### 3.1 Metabolic support index quantifies the benefits derived by organisms in a community

To quantify the benefits derived by the organisms in a community, we computed the metric, Metabolic Support Index (MSI), as described in Methods §2.1.1. MSI essentially quantifies the fraction of an organism’s metabolic network that is “enabled” by the presence of the other organism. An MSI of zero indicates that the organism does not receive any metabolic support from the other, while an MSI of unity indicates that the organism receives all the metabolites required to “enable” the *stuck* reactions. In essence, MSI points to the metabolic exchanges between the organisms.

We calculated MSI for several pairs of organisms, which have been experimentally demonstrated to grow together as a community and where mutual interactions between the organisms have been reported (Table 1). By comparing the MSI of individual organisms, we determined the organism from the pair that derives the maximum benefit. In addition, we also quantified the interactions based on the increase in the number of metabolites produced by one organism in the presence of the other (Supplementary Table 1). Such an increase points to the synergy and the possible metabolic interactions between the organisms. Our observations also point to an increase in metabolic co-operation as reported previously [11].

**Table 1:** MSI to quantify pairwise interactions. The table presents pairwise MSI for examples from the literature. The higher the value of MSI, the better is the benefit the organism derives through this interaction. The references in Column 1 and 2 pertain to the source of the respective GSMMs. The value in bold in each row indicates the MSI of the organism that benefits from the interaction.

Organism A	Organism B	*MSI(A)*	*MSI(B)*	Comments
*Ketogulonicigenium vulgare*[25]	*Bacillus megaterium*[25]	**0.107**	0.0	*B. megaterium* acts as a helper strain for *K. vulgare* by providing additional metabolites [36], [37]
*Clostridium cellulolyticum*[28]	*Clostridium acetobutylicum*[26]	**0.039**	0.001	*C. acetobutylicum* helps *C. cellulolyticum* to grow and metabolize cellulose under non-favourable conditions through metabolic exchanges [38]
*Pichia stipitis*[29]	*Saccharomyces cerevisiae*[27]	**0.063**	0.025	*P. stipitis* benefits from the interaction with *S. cerevisiae* by taking up additional metabolites from the latter (this study)
*Yarrowia lipolytica*[30]	*Cellulomonas fimi*[30]	**0.119**	0.014	*C. fimi* provides additional metabolites to *Y. lipolytica* in a co-culture setup [17]
*Desulfovibrio vulgaris*[30]	*Methanococcus maripaludis*[30]	**0.176**	0.03	*D. vulgaris* benefits from the interaction with *M. maripaludis*[39]

In all the cases considered, organism A (Column 1) derives a higher benefit in the co-culture than organism B (Column 2). These observations are in exact agreement with those made by the experimental studies (Column 5) evaluating the relative biomasses of the two organisms and their ability to co-exist. For instance, we observe that the MSI of *Ketogulonicigenium vulgare* is 0.1, while that of *Bacillus megaterium* is 0. This indicates that no additional pathways have been activated in *B. megaterium*, in the presence of *K. vulgare*. On the other hand, in *K. vulgare*, 41 additional reactions were *visited* in the co-culture (Supplementary Table 1), which were originally *stuck* in the mono-culture. These results indicate the additional metabolic support *K. vulgare* receives from *B. megaterium*. It is interesting to note that this co-culture system has been widely studied for its applications in vitamin C production; these studies also indicate that *B. megaterium* is a helper strain that enhances the growth and proliferation of *K. vulgare* [33], [34], [35].

In another co-culture consisting of *Yarrowia lipolytica* and *Cellulomonas fimi*, we observe that MSI of *Y. lipolytica* (0.119) is almost 10 times that of *C. fimi* (0.014). This is because of the additional 123 reactions in *Y. lipolytica* that have now been *visited*, in the presence of *C. fimi*. The conversion of these *stuck* reactions to *visited* reactions in the co-culture also points to the enrichment in the metabolic capabilities of *Y. lipolytica*, as also observed from the increase in the *scope* size of joint metabolic networks (Supplementary Table 1). These results indicate that *Y. lipolytica* derives the maximum benefit when grown as a co-culture with *C. fimi*. These metabolic exchanges could have led to the increase in its growth in co-culture, as observed in the experimental studies previously reported [17]. Similarly, in all the other cases we have considered, we show that the trends in MSI agree exactly with the results demonstrated experimentally (Table 1).

Next, we calculated the MSI for the co-culture of our interest, consisting of *S. cerevisiae* and *P. stipitis*. Here, we compute a MSI of *P. stipitis* of 0.063, nearly three times higher than that of *S. cerevisiae* (0.025). In addition, we observe 21 reactions in *P. stipitis* that have been enabled in the presence of *S. cerevisiae*. These results indicate that *P. stipitis* benefits from the interaction with *S. cerevisiae*, which we proceeded to verify using growth kinetic experiments.

### 3.2 *In silico* pathway analyses reveals several interesting metabolic exchanges

To investigate the metabolic exchanges between *S. cerevisiae* and *P. stipitis*, we exhaustively enumerated all the pathways on a community metabolic network, using our previously developed algorithm MetQuest (see Methods). MetQuest identifies all possible pathways from a given set of seed metabolites to all the reachable metabolites, whose size is less than or equal to a given cut-off. The pathway so obtained is complete, i.e., it contains all the reactions necessary to produce every metabolite constituting the pathway.

We computed all the pathways starting from D-glucose in *S. cerevisiae* that lead to various metabolites in *P. stipitis*. In total, we observed multiple pathways in *P. stipitis* that were involved in the production of 668 different metabolites (Supplementary Table 2). A closer analysis of these pathways revealed the presence of 45 different exchange metabolites from *S. cerevisiae*. These exchange metabolites were involved in the production of 634 metabolites (Supplementary Table 2). Of these 45 metabolites, we note that acetaldehyde, α-ketoglutarate, ethanol and sorbitol were the most commonly exchanged metabolites since they involved in the production of over 400 metabolites in *P. stipitis* (Supplementary Fig. S1). Further, we checked for the presence of transporters for these 45 metabolites from their GSMMs and identified that 22 metabolites had putative protein transporters, 9 metabolites were transported via proton symport or antiport and the rest 14 were transported via passive transport/diffusion (Supplementary Table 3).

Further, to determine if there were any additional benefits in terms of metabolic exchanges leading to the overall biomass production, we analysed all the pathways from *S. cerevisiae* D-glucose to the amino acids in *P. stipitis*. Amongst the 45 metabolites listed above, we observed a total of 34 exchange metabolites to be involved in the pathways producing different amino acids (Supplementary Table 4). It was interesting to note that of the most commonly exchanged metabolites, acetaldehyde, α-ketoglutarate, ethanol and sorbitol take part in the production of more than ten amino acids.

Several studies previously carried out on this co-culture have demonstrated its ability to produce ethanol from a variety of substrates [21], [22]. However, from our computational predictions, we observed ethanol transfer from *S. cerevisiae* and *P. stipitis*. The ethanol so transferred was involved in multiple pathways, which produced 418 metabolites in *P. stipitis* (Supplementary Table 5). We then decided to experimentally check if ethanol was indeed exchanged between the organisms, as predicted from our pathway analyses. This would help in designing better processes with a predefined harvest time where this co-culture of organisms is used for ethanol production from other carbon sources.

### 3.3 *P. stipitis* exhibits higher cell density in cell-free supernatant of *S. cerevisiae*

**Fig. 3:**
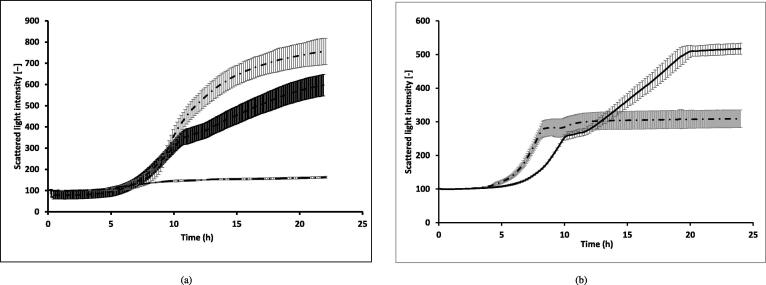
Growth curves: (a) Growth curves of mono- and co-cultures of *P. stipitis* and *S. cerevisiae*. These experiments were carried out in YNB (defined) medium. Here,  represents the co-culture of *P. stipitis* and *S. cerevisiae*,  represents the mono-culture of *P. stipitis* and  represents the mono-culture of *S. cerevisiae*. Note that the growth curve of co-culture exhibits a diauxic pattern and lies between those of mono-cultures of *P. stipitis* and *S. cerevisiae*. (b) Comparison of the growth curve of *P. stipitis* grown on the supernatant of *S. cerevisiae* with that grown in control. This experiment was performed according to the scheme showed in Fig. 2. Here,  represents control and  represents experimental condition. Note the diauxic pattern of growth curve when *P. stipitis* is grown in the supernatant of *S. cerevisiae*. All the experiments were carried out at 30°C and 300 rpm. In both the cases, the growth was monitored online using CGQ (Aquila Biolabs). The experiments were performed in triplicates. Error bars represent the standard deviation values obtained from CGQuant software version 7.3. For more details on methods, see §2.2.3 and §2.2.4.

To check for the existence of mutual interactions between *S. cerevisiae* and *P. stipitis*, we carried out mono- and co-culture growth kinetics experiments with these organisms on a minimal medium containing 10 g/L D-glucose as the carbon source. From the growth curves (Fig. 3a), we observed that the co-culture cell density was in between that of the respective mono-cultures. This provided indications on the ability of the organisms to co-exist. Further, the co-culture showed a diauxic pattern of the growth curve, indicating that a few metabolites from the supernatant may serve as the carbon source.

Next, to identify the organism that benefits from the interactions, we designed and carried out the two-stage experiment. In stage 1, the first organism was grown in a minimal medium consisting of glucose. In stage 2, the supernatant from the first organism was used as the medium to grow the second organism, and the growth was compared with that of the control where sterile distilled water was used instead of the supernatant (refer §2.2.4 for more details). Interestingly, from the growth curves of the stage 2 (Fig. 3b), we observed that at the end of 24 h, *P. stipitis* exhibited a 1.34-fold higher cell density (520) when grown in the supernatant of *S. cerevisiae*, in comparison to that seen in the control (310). In addition to the increased cell density, *P. stipitis* clearly exhibited a diauxic growth pattern, indicating the presence of an alternate metabolite in the supernatant that was used as the carbon source. We did not observe any increase in the cell density of *S. cerevisiae* when grown in the supernatant of *P. stipitis*. This observation also corroborates the higher MSI for *P. stipitis* in the community, indicating that *P. stipitis* derives a higher benefit when grown together with *S. cerevisiae* under these conditions.

### 3.4 *In silico* pathway analyses and isotope labelling experiments confirm ethanol transfer from *S. cerevisiae* to *P. stipitis*

In the next step, we experimentally determined the metabolic exchanges happening between *S. cerevisiae* and *P. stipitis* using the same two-stage experimental setup. We performed HPLC analyses on the samples withdrawn at 0 h and 24 h time points of the second stage, where *P. stipitis* was grown in the supernatant of *S. cerevisiae*. Interestingly, we observed that the concentration of ethanol had reduced from 2.4 g/L (in 0 h) to 0.0 g/L (24 h).

**Fig. 4:**
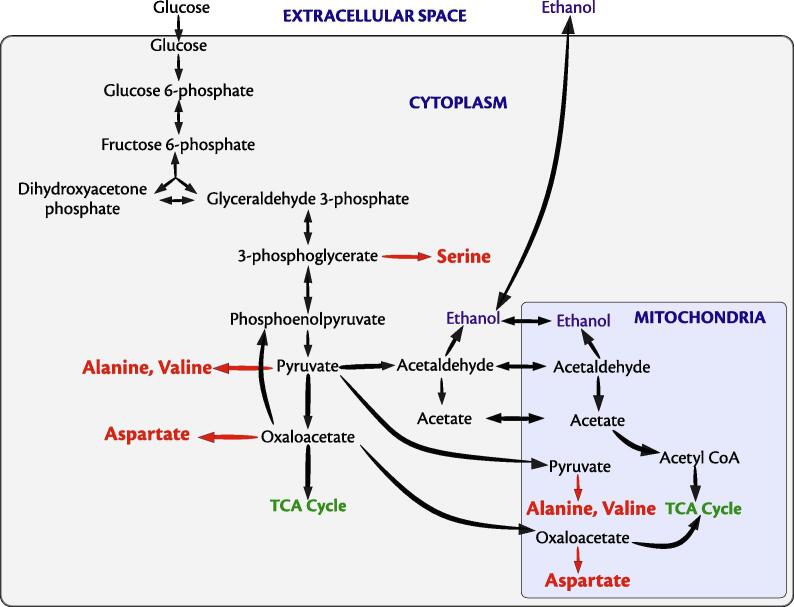
Metabolic network showing the central carbon metabolism in *P. stipitis*. Ethanol is directly involved in the production of amino acids aspartate, alanine and valine, which is produced from oxaloacetate and pyruvate. Through gluconeogenesis pathway, which has been reported to be observed when yeasts grow on non-fermentable carbon sources, ethanol is also involved in the production of serine.

**Fig. 5:**
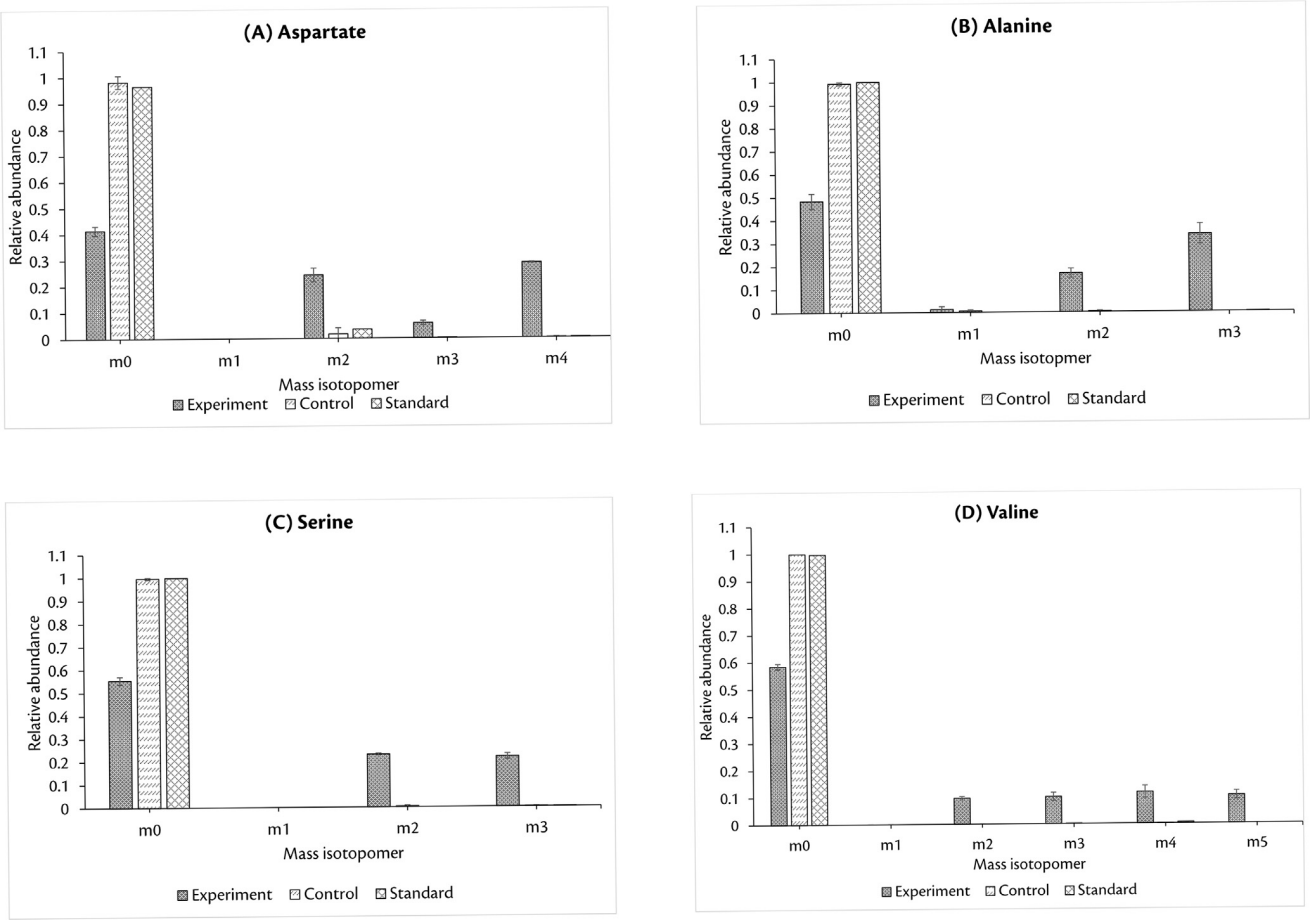
Mass isotopomer distributions of four different amino acids arising from ethanol metabolism. (A) Aspartate, (B) Alanine, (C) Serine, (D) Valine. The relative abundance of heavier isotopomers of amino acids is much higher in comparison to that of control, indicating the uptake of a labelled carbon source. Note that we considered (M-57)+ fragment for our analysis. The three bars indicate the results from the experiment, the control and the standard, as indicated in the legend.

Motivated by the reduction in the ethanol concentration and the predictions of metabolic exchanges from our computational studies (§3.2), we hypothesised that *P. stipitis* was consuming the ethanol produced by *S. cerevisiae*. To confirm this, we studied the ^13^C labelling patterns of amino acids. We used the metabolic network of *S. cerevisiae* [40], [41] to line-out the ethanol metabolism in *P. stipitis* (Fig. 4). As in all yeast, the carbon skeleton of ethanol is incorporated into the TCA cycle [42], while the anaplerotic reaction is the glyoxylate shunt. Hence, one would expect label to be present in aspartate (from oxaloacetate) and glutamate (from ketoglutarate). The malate and oxaloacetate can be converted to pyruvate to fuel gluconeogenesis, which is one of the indispensable pathways when yeasts grow on non-fermentable carbon sources [43]. The incorporation of the C2 of ethanol into the C3 of, e.g., pyruvate (a compartmented metabolite) and later into phosphorylated C3 of glycolysis in the cytosol can be tracked via alanine, valine, and serine, respectively. We checked for the incorporation of ^13^C-labelled carbon in these amino acids specifically in the (M-57)+ fragment of the respective amino acids. Interestingly, in all these amino acids, we observed isotopomers with a higher fraction of ^13^C incorporated (Fig. 5). For instance, from the pool of amino acid aspartate, we find 28% of m4 isotopomer where all the four carbon atoms carry a ^13^C label. In addition, we also noted that there is a negligible fraction of m1 isotopomer in all the four amino acids. Further, we observed that the average carbon labelling (0.39) in these amino acids is significantly higher than that found in the control (0.005), indicating the uptake of a labelled metabolite from the supernatant (Table 2). In addition, the average carbon labelling observed in the amino acids (0.39) was almost equal to the fraction of labelled substrate present in the supernatant (0.42 ± 0.01) indicating that nearly all the observed labels in the amino acids originated from ethanol.

**Table 2:** Average carbon labelling in amino acids.

Amino	Mass of the	Average carbon labelling	
acids	fragment	Experiment	Control
ala	260	0.455	0.003
asp	418	0.449	0.015
ser	390	0.367	0.002
val	288	0.294	0.001
**Average**		0.391	0.005

## 4 Discussion

Microbial co-cultures have been used for several biotechnological applications, where the metabolic capabilities of two different organisms have been exploited. Several recent studies have designed co-cultures with microorganisms that have been genetically modified to exchange metabolites with one another. Moreover, these microbial co-cultures also serve as a models to study the fundamental cell-cell communication and community life in general. There has indeed been a growing interest in studying these microbial co-cultures/communities and identifying the interactions therein.

In this study, we systematically identify the metabolic interactions between the microorganisms in a co-culture by integrating modelling with experiments. To this end, we applied our previously developed graph-theoretic algorithm that operates on the metabolic networks of the microorganisms. We defined a new metric termed *Metabolic Support Index* (MSI) that quantifies the metabolic support each organism receives from the other in a community, in terms of the number of reactions “enabled”. We computed this metric on several co-cultures presented in literature and demonstrated that this metric can correctly identify the beneficiaries from the microbial interactions.

There have been a few studies carried out in the past that incorporate the nutrient conditions and quantify the co-operation between the organisms in a community. These studies identify the synthesising capabilities of “joint metabolic networks” [11], [44] and compute the benefits in terms of the metabolites produced by the individual organisms in a community. Our metric, MSI, on the other hand, determines the number of additional reactions that can be activated in one organism in the presence of other due to the metabolic exchanges. In addition, MSI also gives an idea about the metabolic enrichment arising out of microbial co-operation. Further, the reactions facilitating metabolic exchanges between the organisms can be used as a potential target for over-expression. Such reactions could be used to improve the co-operation between the organisms through the exchange of metabolites. Experimentally, MSI can be easily verified by designing appropriate medium conditions depending on the input seed metabolites.

Further, to integrate the predictions with the experiments, we calculated MSI for a well-known yeast co-culture system [19] consisting of *P. stipitis* and *S. cerevisiae* and identified that *P. stipitis*, with a higher MSI, benefits from this interaction. Moreover, we also identified the pathways spanning the two organisms and identified the set of metabolites that can be exchanged between the two organisms. Interestingly, we also note that our computational analyses indicate some benefit for *S. cerevisiae* (MSI  = 0.025), while experimentally we observe no such benefit. This is because the calculation of MSI involves only the stuck reactions in one organism that have been relieved in the presence of other organisms. Due to the redundancy in metabolic pathways [45], we observe that metabolites synthesised by these stuck reactions are already produced by other reactions in alternate pathways. However, in the presence of other organisms, these stuck reactions get activated and hence contribute to the MSI value. On the other hand, in experiments, these metabolites would be synthesised by *S. cerevisiae* using its alternate pathways since they are a part of essential components of the cells. Hence this benefit is not observed experimentally.

We also performed two-stage experiments using this co-culture and found that: (a) *P. stipitis* benefits from the interaction, as shown by a higher value of MSI compared to that of *S. cerevisiae* and (b) *P. stipitis* consumes the ethanol produced by *S. cerevisiae* as also observed in previous studies. Also, from the labelling studies, it is also interesting to note the complete absence of m1 isotopomer in all the amino acids analysed. Moreover, since the labelling patterns in alanine and valine are different, it can be inferred that both cytosolic and mitochondrial routes of their synthesis were operational [42].

Our two-stage experiment is a simple methodology to establish a *proof-of-concept* for identifying the microbial interactions in a community. Indeed, the dynamics of the organisms in the co-culture may be different from that observed when the organisms are independently cultivated on the cell-free supernatants. Nevertheless, this study provides first glimpse into interactions in microbial communities. Also, deciphering the co-culture dynamics would entail the use of advanced molecular biological techniques. Moreover, isotope labelling patterns of the individual organisms from the co-culture experiments are difficult to obtain. Although recent studies seek to identify labelling patterns in co-culture, these methods either require extraction of a large number of peptides [46] or are restricted to a co-culture of organisms with identical biomass composition [47].

Also, our computational method to determine the relationship between the organisms in a co-culture, captures only the positive interactions between the organisms, as with other graph-based method based on network expansion [11]. Also, alternate metrics may be required to capture the resource allocation benefits, especially in natural communities where microbes have to adapt to the continually changing niche conditions. Further, the pathways identified by MetQuest is an exhaustive set consisting of all possible exchange metabolites. It is important to note that not all these exchange reactions are likely to occur and there may be few exchange metabolites in undetectable quantities. Pruning this set would require additional information such as thermodynamic values and fluxes of reactions. Weighing the pathways based on their importance could help in ranking these exchanges and further improving the power of MSI to predict interactions. Moreover, it is important to note that not all the reactions that have been activated would have a positive effect on the organism. However, our method gives important information about the reactions that have been activated, which can be used to make informed choices while designing microbial consortia.

In sum, this study provides a systematic methodology to understand the interactions in a microbial community by integrating computational and experimental paradigms. A model-integrated approach, combining data from modelling and experiments, allows to identify the complex metabolic interactions possible between the microbes in a co-culture. Our study also underlines the utility of computational analyses to generate testable hypotheses regarding the interactions between various microbes. This generic workflow can be extrapolated to study the metabolic interactions in many microbial communities. For instance, in a community of three organisms, a simple weighting scheme could be followed using the set of stuck reactions. This unique set, relieved by two individual organisms can be suitably weighed by comparing with the common set of reactions relieved by both the organisms. Multiple pair-wise interactions within the larger communities can be compared to identify how different organisms interact with one another. Overall, our integrated workflow can pave the way for rationally designing and engineering microbial consortia tailored towards specific industrial applications.

## Declaration of interests

None.

## CRediT authorship contribution statement

**Aarthi Ravikrishnan:** Conceptualization, Data curation, Formal analysis, Validation, Funding acquisition, Investigation, Methodology, Writing - original draft, Writing - review & editing. **Lars M. Blank:** Conceptualization, Formal analysis, Funding acquisition, Investigation, Methodology, Supervision, Writing - review & editing. **Smita Srivastava:** Conceptualization, Formal analysis, Funding acquisition, Investigation, Methodology, Supervision, Writing - review & editing. **Karthik Raman:** Conceptualization, Formal analysis, Funding acquisition, Investigation, Methodology, Supervision, Writing - review & editing.
