# Supplementary material for: Investigating metabolic interactions in a microbial co-culture through integrated modelling and experiments
Source: Comput Struct Biotechnol J. 2020 Mar 30;18:1249–58. doi: 10.1016/j.csbj.2020.03.019 (PMC7286961; doi:10.1016/j.csbj.2020.03.019)
Supplement: Supplementary file 1 [file mmc1.pdf]

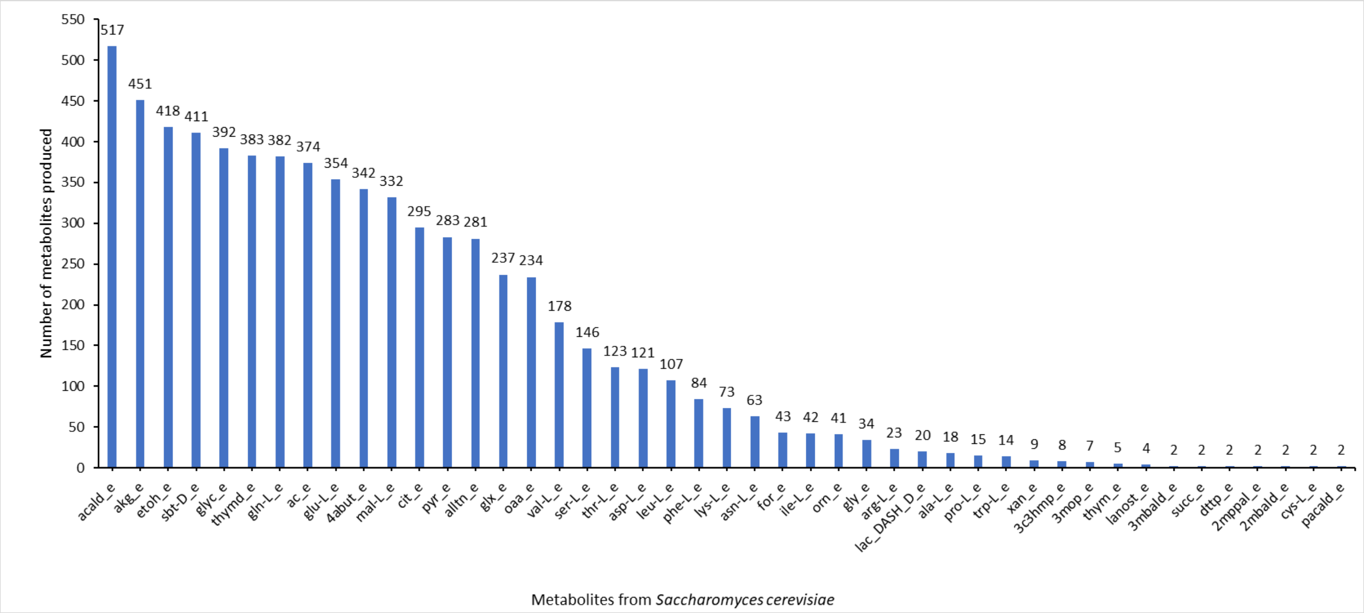

Supplementary Figure S1: This bar chart shows the participation of metabolites from *S. cerevisiae* in the pathways leading to the production of various metabolites in *P. stipitis.* x axis shows the metabolites from *S. cerevisiae,* y axis shows the number of metabolites in *P. stipitis* in whose pathway exchange metabolites from *S. cerevisiae* is involved, the number on the bar represents the exact number of metabolites in *P. stipitis* whose pathways have an exchange metabolite. It is interesting to note that acetaldehyde, alpha ketoglutarate and ethanol are the most commonly exchanged metabolites, in terms of the number of pathways they are involved in, while a few others such as succinate (succ_e), phenylacetaldehyde (pacald_e) help in the production of only a few metabolites. The nomenclature of the metabolites follows those from the genome-scale model of *S. cerevisiae i*MM904.

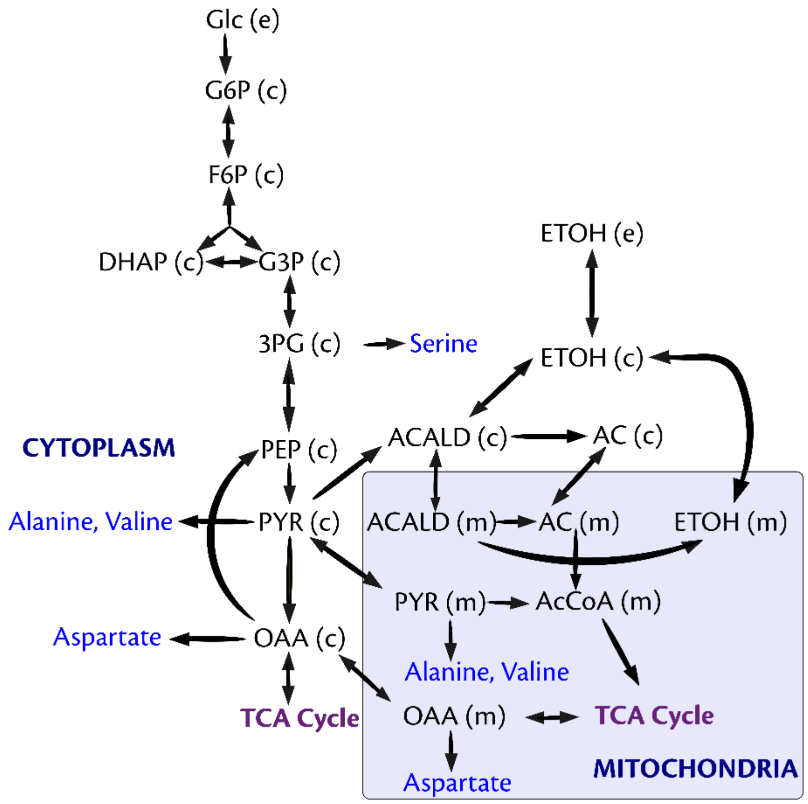

CENTRAL CARBON METABOLISM

Supplementary Figure S2: Metabolic network showing the ethanol metabolism in *P. stipitis*. Ethanol is directly involved in the production of amino acid Aspartate, Alanine and Valine, which is produced from oxaloacetate and pyruvate. Through gluconeogenesis pathway, which has been reported to be observed when yeasts grow on nonfermentable carbon sources, ethanol is also involved in the production of serine.

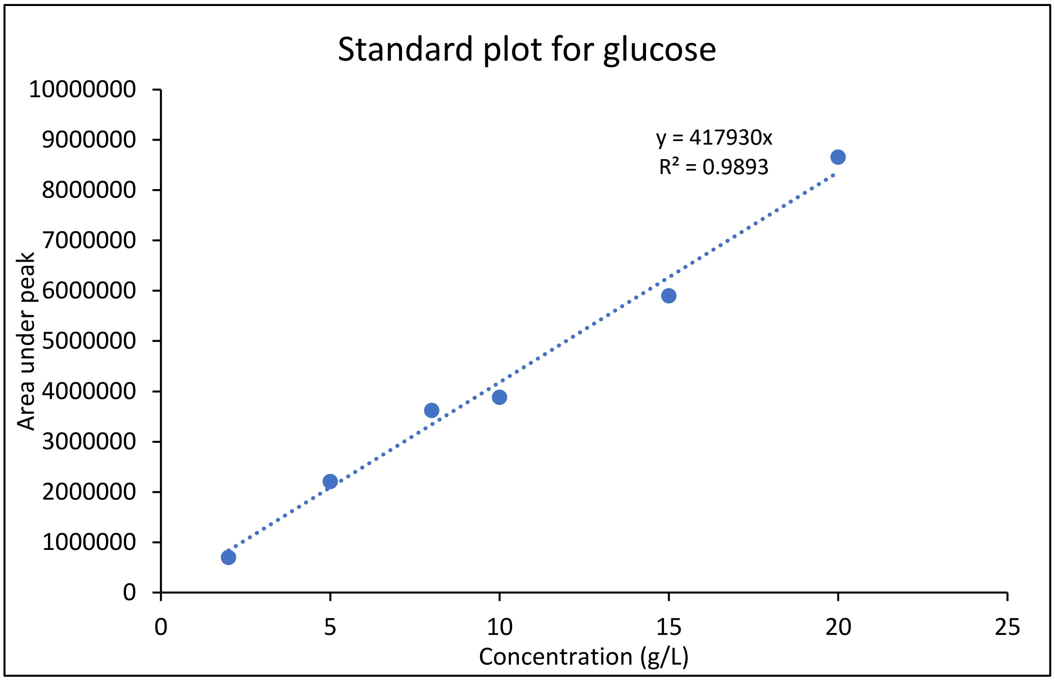

Supplementary Figure S3: Standard plot for glucose for HPLC analysis

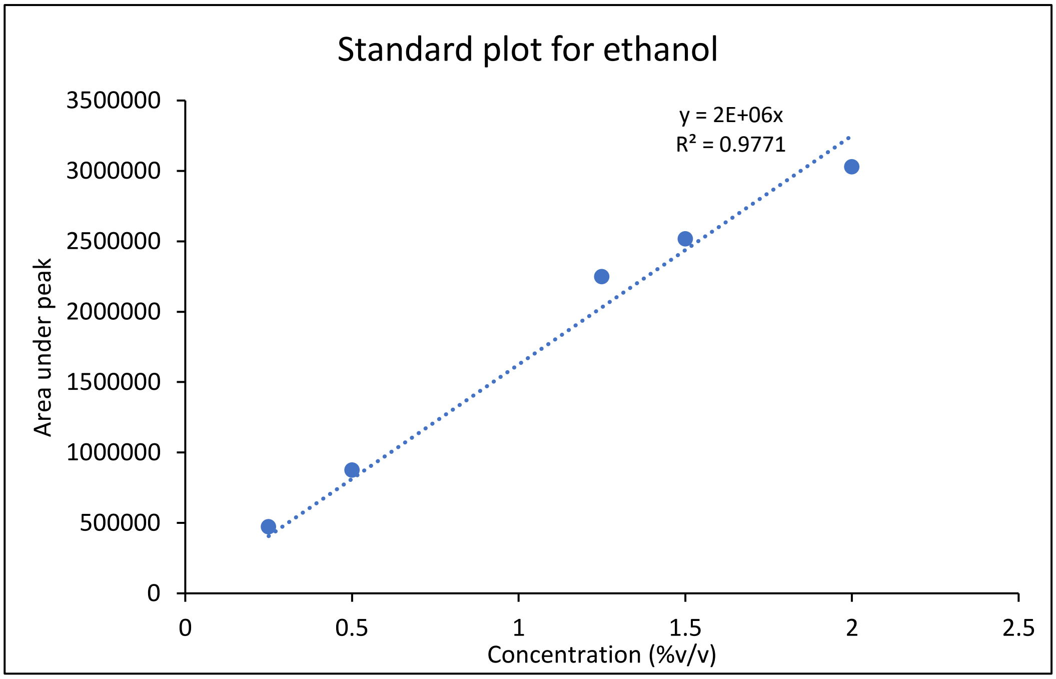

Supplementary Figure S4: Standard plot for ethanol for HPLC analysis
